# Brain MRI volumetry and atrophy rating scales as predictors of amyloid status and eligibility for anti-amyloid treatment in a real-world memory clinic setting

**DOI:** 10.1007/s00415-024-12853-9

**Published:** 2024-12-21

**Authors:** A. Zilioli, A. Rosenberg, R. Mohanty, A. Matton, T. Granberg, G. Hagman, J. Lötjönen, M. Kivipelto, E. Westman

**Affiliations:** 1 Department of Neurology, University-Hospital of Parma, Parma, Italy; 2 Population Health Unit, Finnish Institute for Health and Welfare, Helsinki, Finland; 3 Division of Clinical Geriatrics, Center for Alzheimer Research, Department of Neurobiology, Care Sciences and Society, Karolinska Institutet, Huddinge, Stockholm, Sweden; 4 Department of Clinical Neuroscience, Karolinska Institutet, Stockholm, Sweden; 5 Department of Neuroradiology, Karolinska University Hospital, Stockholm, Sweden; 6 Theme Inflammation and Aging, Karolinska University Hospital, Stockholm, Sweden; 7 Institute of Public Health and Clinical Nutrition, University of Eastern Finland, Kuopio, Finland; 8 Ageing Epidemiology Research Unit, School of Public Health, Imperial College London, London, UK; 9 Combinostics Oy, Tampere, Finland

**Keywords:** Neuroimaging, Brain volumetry, Atrophy rating scales, Anti-amyloid treatment eligibility

## Abstract

Predicting amyloid status is crucial in light of upcoming disease-modifying therapies and the need to identify treatment-eligible patients with Alzheimer’s disease. In our study, we aimed to predict CSF-amyloid status and eligibility for anti-amyloid treatment in a memory clinic by (I) comparing the performance of visual/automated rating scales and MRI volumetric analysis and (II) combining MRI volumetric data with neuropsychological tests and APOE4 status. Two hundred ninety patients underwent a comprehensive assessment. The cNeuro cMRI software (Combinostics Oy) provided automated computed rating scales and volumetric analysis. Amyloid status was determined using data-driven CSF biomarker cutoffs (Aβ42/Aβ40 ratio), and eligibility for anti-Aβ treatment was assessed according to recent recommendations published after the FDA approval of the anti-Aβ drug aducanumab. The automated rating scales and volumetric analysis demonstrated higher performance compared to visual assessment in predicting Aβ status, especially for parietal-GCA (AUC = 0.70), MTA (AUC = 0.66) scores, hippocampal (AUC = 0.68), and angular gyrus (AUC = 0.69) volumes, despite low global accuracy. When we combined hippocampal and angular gyrus volumes with RAVLT immediate recall and APOE4 status, we achieved the highest accuracy (AUC = 0.82), which remained high even in predicting anti-Aβ treatment eligibility (AUC = 0.81). Our study suggests that automated analysis of atrophy rating scales and brain volumetry outperforms operator-dependent visual rating scales. When combined with neuropsychological and genetic information, this computerized approach may play a crucial role not only in a research context but also in a real-world memory clinic. This integration results in a high level of accuracy for predicting amyloid-CSF status and anti-Aβ treatment eligibility.

## Introduction

Alzheimer’s disease (AD) is the most prevalent cause of dementia and an increasing public health concern [1]. It has been estimated that the growing aging population and longer life expectancy could lead to a fourfold increase in AD cases by 2050. This anticipated rise may require adjustments in public health planning and resource allocation [2, 3].

Given this expected increase, it is crucial to have tools that can predict AD-related changes, especially considering the forthcoming availability of disease-modifying therapies and the need to identify and monitor treatment-eligible patients [4].

While cerebrospinal fluid (CSF) assessment is a standard method for obtaining a biological diagnosis of AD, its invasiveness and the need for specialized facilities make it challenging to implement on a large scale, especially given the disease’s increasing impact [5].

In this context, structural neuroimaging is one of the most reliable methods for diagnosing cognitive disorders.

It provides essential data for ruling out the secondary forms of dementia and facilitates diagnostic suspicion toward primary dementias by analyzing atrophy patterns and cerebrovascular findings [6]. Structural imaging has been widely employed for identifying AD-related changes [7]. In this field, visual rating scales assessing the regional atrophy of brain regions, such as medial temporal, parietal, and frontal areas, have been incorporated into routine clinical practice [8–11].

Despite their widespread use, the ability to discriminate between patients affected by AD and other types of dementias has been infrequently investigated, leading to conflicting results. While the medial temporal atrophy (MTA) scale has demonstrated high accuracy in diagnosing AD [12], the scale shows inconsistency in predicting AD-related pathology in other studies [13, 14]. Conversely, parietal atrophy (PA) exhibits high diagnostic accuracy within early-onset cohorts [15]. Recently, the anteroposterior index (API), analyzing the posterior regional pattern of atrophy in AD, has been proposed, showing higher accuracy compared to previous scales [16].

These disparities could be related to the different characteristics of the cohorts but also to the intrinsic subjectivity of visual rating assessment, depending on reader interpretation and adequate expertise. Interest in developing automatically computed rating scales has amplified due to the need to address certain limitations [17]. Nevertheless, the semi-quantitative nature of these scales presents a constraint in detecting subtle brain changes, particularly in the early stages of pre-dementia and cognitive disorders [18]. Volumetric analysis of brain regions might partially address this constraint. Although more confined to a research setting, quantitative assessment can be crucial in assessing the severity and patterns of atrophy [19]. The development of automatic methods in volumetric analysis has marked a significant improvement in terms of time efficiency compared to manual or semi-automatic tools, laying the foundation for potential implementation in clinical settings [20]. However, studies analyzing the potential of automatic analysis in identifying amyloid status in a naturalistic memory clinic, where various cognitive disorders are encountered, still need to be included.

This study utilized the cNeuro cMRI software [17]. This tool provides automated computed rating scales and volumetric analysis. Although initially employed in research settings [21, 22], it is now used in clinical settings for routine assessments, showcasing its potential to bridge research and clinical applications.

This study aimed to predict CSF-amyloid status in a naturalistic memory clinic, primarily comprising patients who were referred to diagnostic evaluation for cognitive impairment, using the following methods: (I) comparing the predictive performances of visual rating scales, automatically computed rating scales, and volumetric analysis; and (II) developing a model that combines volumetric data with neuropsychological and genetic information to predict CSF-amyloid status accurately. Our analysis also focused on assessing the accuracy in predicting eligibility for monoclonal antibody treatment in AD.

## Methods

### Participants and study design

The participants were recruited at the Memory Clinic, Karolinska University Hospital in Solna, Sweden. The specialized outpatient clinic provides comprehensive evaluations for individuals experiencing cognitive concerns. Notably, all diagnostic assessments were conducted promptly within a week, following a “fast track model” described in recent work [23]. The diagnostic evaluation includes various components: a comprehensive medical and neurologic examination, a detailed medical history gathered from both the patient and informants, a comprehensive neuropsychological assessment, blood chemistry analyses, brain MRI, APOE genotyping, and CSF biomarker analysis (Aβ42, Aβ42/40 ratio, phosphorylated (p)-tau181, total (t)-tau, and neurofilament light chain levels). The diagnostic process adheres to the criteria outlined in the Diagnostic and Statistical Manual of Mental Disorders (Fifth Edition) (DSM-V). In addition, the clinic employs the International Classification of Diseases, Tenth Revision (ICD-10) coding system.

The patients affected by cognitive complaints were enrolled in the study between April 2018 and February 2021. Referrals for patients with significant medical conditions (e.g., psychiatric, cardiovascular, or cerebrovascular disease, depression, or cancer) were accepted once the referring clinic confirmed the stability of the condition and its related treatment for a minimum of 3 months.

Between April 2018 and February 2021, 290 consecutive patients underwent a comprehensive assessment, including CSF and MRI analyses with visual rating scales, computed automatic rating scales, and volumetric evaluation.

We assessed and combined the performances of visual and computed rating scales, volumetric analysis, APOE4, and neuropsychological tests in predicting CSF-amyloid status (Aβ42/Aβ40 ratio) in the entire cohort. In an additional investigation, we limited the analysis to evaluate the performance in predicting eligibility for anti-Aβ Treatment.

The Karolinska University Hospital electronic database and biobank for clinical research (GEDOC) and this study have received ethical approval (Regional Ethical Review Board in Sweden). All procedures were performed with the participants’ informed consent.

### Neuroimaging assessment

Patients underwent 3 Tesla brain MRI scans with a GE Medical Systems Discovery MR750 scanner (GE HealthCare, Milwaukee, WI, USA) with a standard clinical head coil. A routine protocol was followed, including sagittal T1-weighted 3D BRAVO (slice thickness: 1 mm; repetition time: 8.16 ms; echo time: 3.18 ms), sagittal T1-weighted FLAIR (slice thickness: 4 mm; repetition time: 1680 ms; echo time: 26.53 ms), sagittal T2-weighted FLAIR 3D CUBE (slice thickness: 1.2 mm; repetition time: 8000 ms; echo time: 116 ms), axial T2-weighted PROPELLER (slice thickness 4 mm; repetition time: 4000 ms; echo time: 86 ms), axial DWI (slice thickness: 4 mm; repetition time: 3501 ms; echo time: 62 ms), and axial SWI 3D (slice thickness: 2 mm; repetition time: 30 ms; echo time: 20 ms).

Experienced neuroradiologists carefully evaluated the scans and visually assessed several parameters during routine clinical practice.

**Fig. 1:**
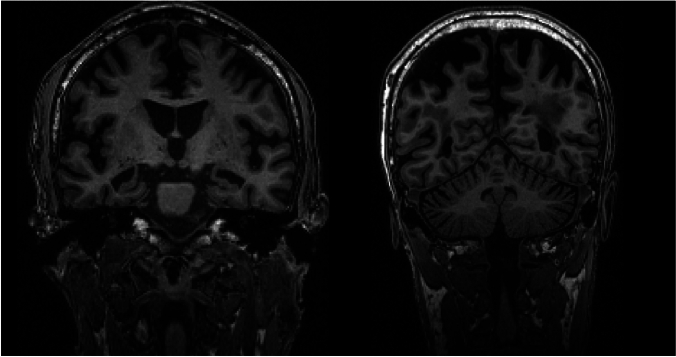
Coronal 3D T1-weighted brain MRI scans with MTA = 3 bilaterally (left panel) and with GCA-P = 3 (right panel)

Medial temporal lobe atrophy (MTA) was assessed using the Schelten’s scale (0–4; higher scores indicate greater atrophy) [8], global cortical atrophy (GCA) was evaluated on a scale of 0–3 (higher scores indicate greater atrophy) [9], posterior atrophy (PA) was measured using the Koedam scale (0–3; higher scores indicate greater atrophy) [10], and white matter hyperintensities (WMH) were assessed utilizing the Fazekas scale (0–3; higher scores indicate a greater cerebrovascular burden within the white matter) [24] according to a standardized protocol [11] (Fig. 1). Complete MRI reports were not available to assess potential imaging-related contraindications such as microbleeds.

**Fig. 2:**
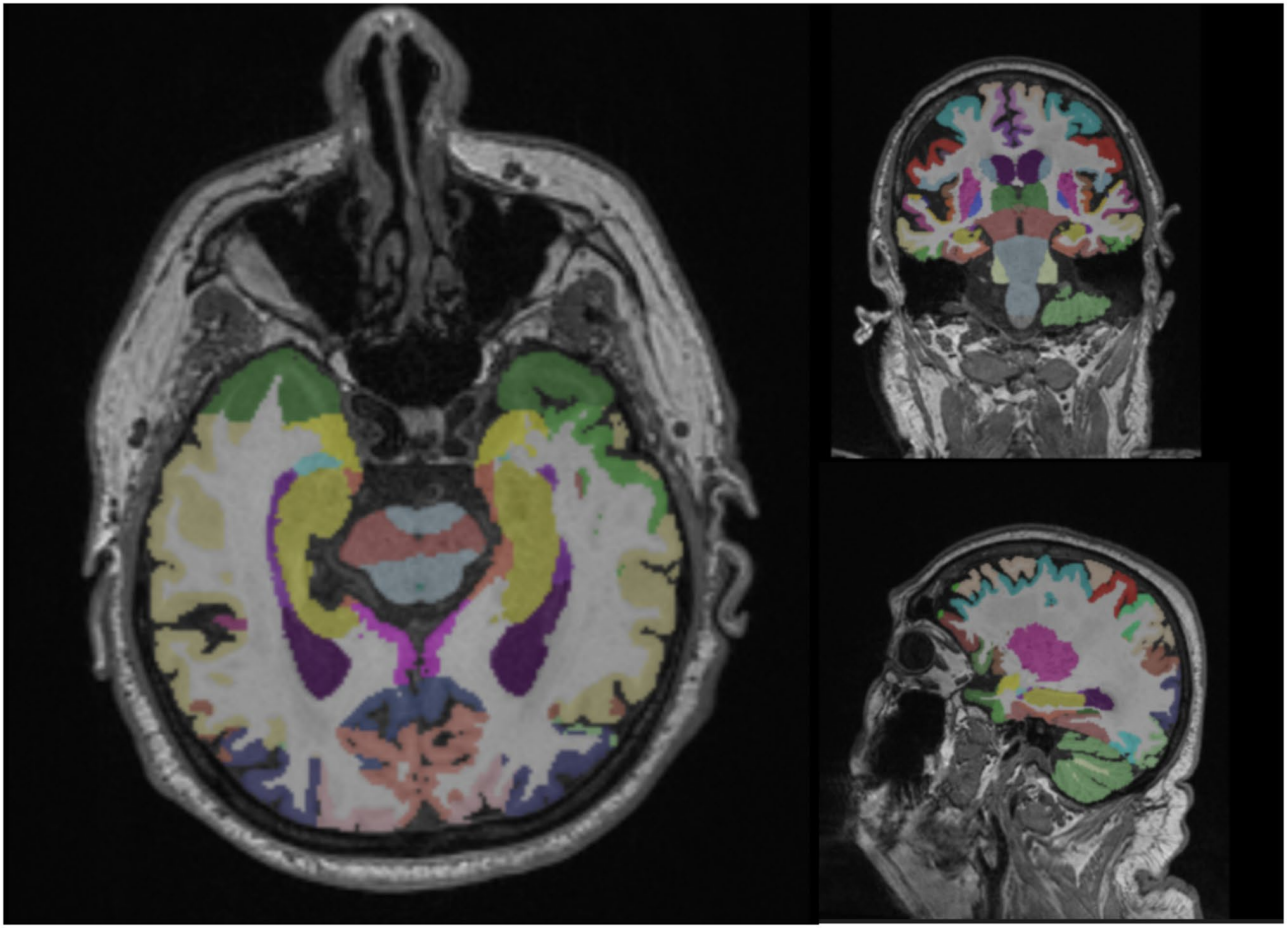
Illustration of the structural segmentation from T1 3D images using cNeuro cMRI software

Furthermore, we utilized the cNeuro cMRI software (Combinostics Oy, Finland, www.cneuro.com/cmri/) [17]. This software allowed automated analysis of regional brain volumes (cortical and subcortical) and WMH, using 3D T1-weighted and T2-weighted FLAIR sequences and a multi-atlas method to segment the data into 133 regions [25] (Fig. 2). To ensure accurate individual comparisons, all volumes were standardized by normalizing them for head size using a brain size scaling factor, age, and gender. The automated image quantification tool also generated estimates of standard rating scales, including MTA, Fazekas score, and global and regional GCA. This allowed for a more detailed evaluation of regional atrophy, specifically in the frontal (GCA-F), parietal (GCA-P), and occipital (GCA-O) regions. The validity of this automated analysis was previously established [22].

### Cerebrospinal fluid analysis

Cerebrospinal fluid (CSF) samples were collected under sterile conditions using polypropylene tubes (Medicarrier art. no. 67741) and subsequently analyzed at the Karolinska University Hospital Laboratory as part of the diagnostic evaluation process. Before August 21, 2019, the concentrations of Aβ42, Aβ40, phosphorylated tau 181 (p-tau181), total tau (t-tau), and neurofilament light chain (NFL) were determined using commercially available Innotest sandwich enzyme-linked immunosorbent assays (Fujirebio Europe, Ghent, Belgium). On August 22, 2019, CSF samples were analyzed using the Lumipulse G-series (Fujirebio Europe Ghent, Belgium), a fully automated chemiluminescent enzyme immunoassay platform. Previous studies have demonstrated a strong agreement between the two analytical methods [26]. To differentiate between normal and pathological values, data-driven CSF biomarker cutoffs (Aβ42/Aβ40 ratio × 10) were determined. For Innotest, amyloid positivity was defined as a value < 0.60, and for Lumipulse, it was defined as a value < 0.86 [23].

### Blood biomarkers analysis

Patients underwent serum screening to assess lipid risk, analyzing total cholesterol, LDL-cholesterol levels, HDL-cholesterol levels, and serum homocysteine. In addition, genetic risk was evaluated by including the analysis of the APOE gene.

### Cognitive evaluation

The neuropsychological assessment included the Montreal Cognitive Assessment (MoCA) [27] for the evaluation of global function, the Rey Auditory Verbal Learning Test (RAVLT) with immediate and delayed recall [28] for memory assessment, the Rey Complex Figure Test immediate retention [29] for visuospatial abilities and Wechsler Adult Intelligence Scale (WAIS) 4th edition coding subtest for attention/speed functions [30].

### Eligibility for anti-Aβ treatment

To assess potential real-life eligibility for anti-Aβ treatment, we followed recent appropriate use recommendations published after the FDA approved the initial anti-Aβ drug aducanumab [31]. Patients were deemed eligible if they satisfied the following criteria: (1) clinical diagnosis of AD-type dementia or MCI (with no evidence of non-AD neurologic disorder); (2) MMSE 21–30 (or MoCA 17–30); (3) available MRI; (4) absence of anticoagulant treatment; and (5) CSF profile consistent with AD (A+, abnormal Aβ42/Aβ40 ratio) [23]. Detailed methods to assess anti-Aβ treatment eligibility are described elsewhere [23].

### Statistical analysis

All quantitative data are presented as means and standard deviations (SD). We assessed differences in demographics, education, and cognition using the Kruskal–Wallis test. We employed the ANCOVA test to explore variations between the amyloid-positive and amyloid-negative groups, specifically analyzing visual and computed rating scales and volumetric data. To calculate the area under the curve (AUC) and the accuracy, we utilized neuroimaging data (visual rating scales, automated computed scales, and volumetric assessment), neuropsychological values, and APOE4 status as independent variables in a binomial logistic regression model, with amyloid status (normal/abnormal based on Aβ42/Aβ40 ratio) as the dependent variable. For each test, the cutoff value associated with the highest Youden Index was chosen as the reference.

Initially, we developed a predictive model focusing only on the most predictive structural neuroimaging data. The two regions, one for the medial temporal and one for cortical areas (averaging left and right), which exhibited the highest accuracy, were combined, and one point was assigned if the values fell below the reference cutoff, obtaining a total score with a value ranging from 0 to 2.

Furthermore, we aimed to create a model that combined neuroimaging data with neuropsychological tests and APOE4 status. One point was assessed for the most predictive neuropsychological data if the value was under the value chosen as reference (based on the highest Youden Index) and one point for APOE4 status; the points for the neuroimaging data were assigned as described above.

The predictive score (a total score ranging from 0 to 4) underwent testing in a binomial logistic regression model to evaluate (1) sensitivity and specificity values, (2) positive and negative predictive values (PPV and NPV), and (3) receiver operating characteristic (ROC) curve analyses, including their respective AUC and 95% confidence intervals. Only data from regions and tests displaying the highest accuracy were presented regarding volumetric and neuropsychological data.

DeLong’s test was employed to analyze potential statistical differences among AUCs (e.g., visual rating scale vs automatic assessment).

Data-driven CSF biomarker cutoffs (Aβ42/Aβ40 ratio) were determined using Gaussian mixture modeling (GMM) as highlighted in previous studies [23] for a complete description of GMM applied to data-driven CSF biomarkers. The analyses were conducted using the open-source statistical software Jamovi v. 2.3.21.0.

## Results

### Study population characteristics

Our study included 290 patients with an average age of 59.8 (SD: ± 7.0) years. The patients had, on average, 13.6 years of education (± 3.3), and 167 patients (57.6%) were females. The average MoCA score was 22.46 (± 5.2). Of all patients, 92 (31.7%) were amyloid-positive. Table 1 illustrates the demographic characteristics of amyloid-positive and -negative groups.

**Table 1:** Summary of population

	*N*	Amyloid-CSF negative (*N* = 198)	Amyloid-CSF positive (*N* = 92)	*p* value
Age (± SD)	290	58.4 (± 7.1)	62.8 (± 5.8)	< 0.001
Sex (% female)	290	58.5%	55.4%	0.614
Education (± SD)	267	13.7 (± 3.4)	13.6 (± 3.0)	0.745
MoCA (± SD)	282	23.4 (± 4.6)	20.3 (± 5.7)	< 0.001
MMSE (± SD)	232	26.5 (± 3.9)	24.8 (± 4.5)	0.004
Clinical diagnosis	290	67.1% SCI, 19.1% MCI, 13.6% dementia	22.8% SCI, 25% MCI, 52.1% dementia	< 0.001
Apoε4 carrier	280	29.8%	69.6%	< 0.001
RAVLT immediate recall score (± SD)	254	44.6 (± 12.7)	35.8 (± 13.4)	< 0.001

There were no differences in sex (*p* = 0.614) and education (*p* = 0.745) between the two groups. However, the amyloid-positive patients were significantly older (*p* < 0.001) and had worse cognition (*p* < 0.001 for MoCA score) compared to the amyloid-negative group. Within the amyloid-positive group, 21 patients (22.8%) had subjective cognitive impairment (SCI), 23 (25.0%) had mild cognitive impairment (MCI), and 48 (52.1%) had dementia. All patients affected by MCI and dementia exhibited relatively typical forms of memory-predominant AD except for one patient who was diagnosed with an atypical variant (posterior cortical atrophy).

In the group of amyloid-negative patients, 133 (67.1%) patients were considered to have SCI, 38 (19.1%) MCI, and 27 (13.6%) dementia.

Most diagnoses were cognitive disorders of unspecified etiology (*N* = 142), followed by psychiatric disorders (*N* = 20), vascular disorders (*N* = 7), alcohol-related (*N* = 6), frontotemporal dementia (*N* = 5), and other cognitive disorders.

### Visual rating scales (neuroradiologist) and prediction of amyloid-CSF status

In the amyloid-positive group, the average MTA score was 0.97 (range 0–4), with specific scores of 0.94 for the left side and 1.01 for the right side. The mean PA score was 0.80 (range 0–3), the mean Fazekas score was 1.07 (range 0–3), and the mean GCA score was 1.17 (range 0–3). In the amyloid-negative group, the average MTA score was 0.74, consisting of scores of 0.71 for the left side and 0.77 for the right side. The mean PA score was 0.62, the mean GCA score was 0.86, and the mean Fazekas score was 1.00. Statistical analysis revealed significant differences between the amyloid-positive and amyloid-negative groups for MTA (*p* = 0.014) and GCA (*p* < 0.001). However, no statistically significant differences were observed for PA (*p* = 0.063) and Fazekas scores (*p* > 0.1). In the prediction of amyloid-CSF status, GCA exhibited the highest accuracy with an AUC of 0.62. In contrast, overall MTA and PA accuracy were numerically lower, with AUC values of 0.60 and 0.58, respectively.

### Computed automatic rating scales and prediction of amyloid-CSF status

The mean computed MTA score in the amyloid-positive group was 0.82, with specific scores of 0.83 for the right side and 0.81 for the left side. In addition, the mean PA was 1.09, the mean frontal-GCA was 1.17, and the mean occipital-GCA was 0.94. The mean Fazekas computed score was 0.68. The mean computed MTA score in the amyloid-negative group was 0.45, with specific scores of 0.47 for the right side and 0.44 for the left side. The mean PA was 0.58, the mean frontal-GCA was 0.68, and the mean occipital-GCA was 0.55. The mean Fazekas computed score was 0.68. It is worth noting that all scores exhibited statistically significant differences between the two groups (*p* < 0.001), except for the Fazekas score (*p* > 0.1). Regarding predicting amyloid cerebrospinal fluid (CSF) status, the PA score achieved the highest accuracy with an AUC of 0.70. Meanwhile, MTA, frontal-GCA, and occipital-GCA displayed slightly lower accuracy, with AUC values of 0.66, 0.65, and 0.64, respectively. Comparing AUC, computed MTA and PA were significantly more accurate than the ratings performed by neuroradiologists for the same scales (*p* < 0.05). Table 2 summarizes the results of visual and computed rating scales. Table 3 illustrates the differences in accuracy between visual ratings performed by neuroradiologists and computed rating scales.

**Table 2:** Mean values of visual (neuroradiologists) and computed rating scales in the amyloid-positive and negative groups

	Amyloid-CSF negative	Amyloid-CSF positive	*p* value
MTA visual	0.74 (± 0.71)	0.97 (± 0.71)	**0.014**
MTA computed	0.45 (± 0.73)	0.82 (± 0.86)	**< 0.001**
PA visual	0.62 (± 0.70)	0.80 (± 0.70)	0.063
PA computed	0.58 (0.78)	1.09 (± 0.9)	**< 0.001**
GCA visual	0.86 (± 0.63)	1.17 (± 0.68)	**< 0.001**
Frontal-GCA computed	0.68 (± 0.86)	1.17 (1.01)	**< 0.001**
Occipital-GCA computed	0.55 (± 0.73)	0.94 (± 0.82)	**< 0.001**
Fazekas visual	1.00 (± 0.70)	1.07 (± 0.63)	> 0.1
Fazekas computed	0.68 (± 0.91)	0.68 (± 0.91)	> 0.1

In bold, p-values ≤ 0.05

**Table 3:** Direct comparison among the predictive accuracy of visual and automatic rating scales for the amyloid-CSF status

Rating scales	AUC	CI 95%	*p* value
V-MTA	0.603	0.534–0.671	**0.048**
C-MTA	0.663	0.596–0.729	
V-PA	0.583	0.508–0.657	**0.050**
C-PA	0.669	0.602–0.735	
V-GCA	0.629	0.560–0.697	
C-Frontal-GCA	0.653	0.586–0.719	> 0.05
C-Occipital-GCA	0.648	0.577–0.718	
V-Fazekas	0.555	0.488–0.614	
C-Fazekas	0.496	0.430–0.576	> 0.05

*V* visual, *C* computed

*p* values show the statistical significance among different AUCs

In bold, p-values ≤ 0.05

### Volumetric analysis and prediction of amyloid-CSF status

In our analysis of various brain regions using the automated image quantification tool, the average hippocampus and angular gyrus volumes demonstrated the highest accuracy in predicting amyloid-positive status for the medial temporal and cortical areas, respectively. The mean hippocampal volume was 6.88 (± 0.95) ml for the amyloid-negative group and 6.33 (± 0.92) ml for the amyloid-positive group. Conversely, the mean angular gyrus volume was 8.82 (± 1.11) ml for the amyloid-negative group and 7.96 (± 1.26) ml for the amyloid-positive group. Both regions exhibited significant differences between the two groups (*p* < 0.001). The hippocampal volume yielded an AUC of 0.68, with a cutoff value of < 6.94 ml, demonstrating the highest Youden Index, resulting in a sensitivity of 79.3 and a specificity of 52.5%. The angular gyrus achieved an AUC of 0.69, with a cutoff value of < 7.80 ml, showing a sensitivity of 47.8% and a specificity of 84.3%. When we combined these two regions into a single predictive model, assigning one point for each pathological value, we observed an enhanced accuracy (AUC = 0.72). Using a cutoff of ≥ 1, we achieved a sensitivity of 68.9% and a specificity of 50.5%. With a cutoff of ≥ 2, the sensitivity was 38.0%, and the specificity was 86.3% (see Table 4).

**Table 4:** Accuracy in predicting the amyloid-CSF status of the volumetric assessment and the predictive model comprehensive of imaging, neuropsychological, and genetic data

	Sensitivity (%)	Specificity (%)	PPV (%)	NPV (%)	AUC (CI 95%)
Amyloid-positive status (*N* = 92)					
Volumetric assessment (hippocampus and angular gyrus)					
≥ 1	86.9	50.5	44.9	89.2	0.722 (0.665–0.778)
≥ 2	38.0	86.3	56.4	75.0	
Predictive model (hippocampus–angular gyrus–RAVLT immediate–Apoε4 status)					
≥ 1	*96*	*27.0*	*36.7*	*93.8*	0.821 (0.770–0.871)
≥ 2	86.6	67.6	54.1	92.0	
≥ 3	52.0	91.7	73.5	81.2	
≥ 4	14.6	96.4	64.7	71.9	
Anti-Aβ treatment eligibility (*N* = 45)					
Volumetric assessment (hippocampus and angular gyrus)					
≥ 1	90.9	43.7	22.9	96.3	0.700 (0.629–0.770)
≥ 2	38.6	81.9	28.3	87.8	
Predictive model (hippocampus–angular gyrus–RAVLT immediate–APOE4 status)					
≥ 1	100	23.9	21.2	100	0.818 (0.761–0.874)
≥ 2	93.0	57.8	31.2	97.5	
≥ 3	58.1	85.1	44.6	90.9	
≥ 4	18.6	95.6	47.0	85.1	

When statistically comparing the AUC values, hippocampal and angular gyrus volumes were significantly more accurate than MTA and PA obtained by neuroradiologist (*p* < 0.05).

### Neuropsychological tests and APOE as predictors of amyloid-CSF status

In our study, the RAVLT immediate recall demonstrated the highest accuracy in predicting amyloid-CSF status among the tests utilized. The mean value for this test was 44.6 (± 12.7) points for the amyloid-negative group and 35.8 (± 13.4) points for the amyloid-positive group. Statistically significant differences were observed between these two groups (*p* < 0.001). For the prediction of amyloid-CSF status, the accuracy of the RAVLT immediate recall yielded an AUC of 0.69. The cutoff value with the highest Youden Index (< 42 points) achieved a sensitivity of 67.0% and a specificity of 71.7%. Regarding APOE4 status, 29.8% of amyloid-negative patients carried this allele; in the amyloid-positive group, this percentage reached 69.6%. Statistically significant differences were evident between the two groups (*p* < 0.001). The APOE4 allele exhibited an AUC of 0.69, with a sensitivity of 70.1% and a specificity of 69.6%.

Combining RAVLT performance with APOE4 genotype yields an AUC of 0.74.

### Predictive model of amyloid-CSF status

**Fig. 3:**
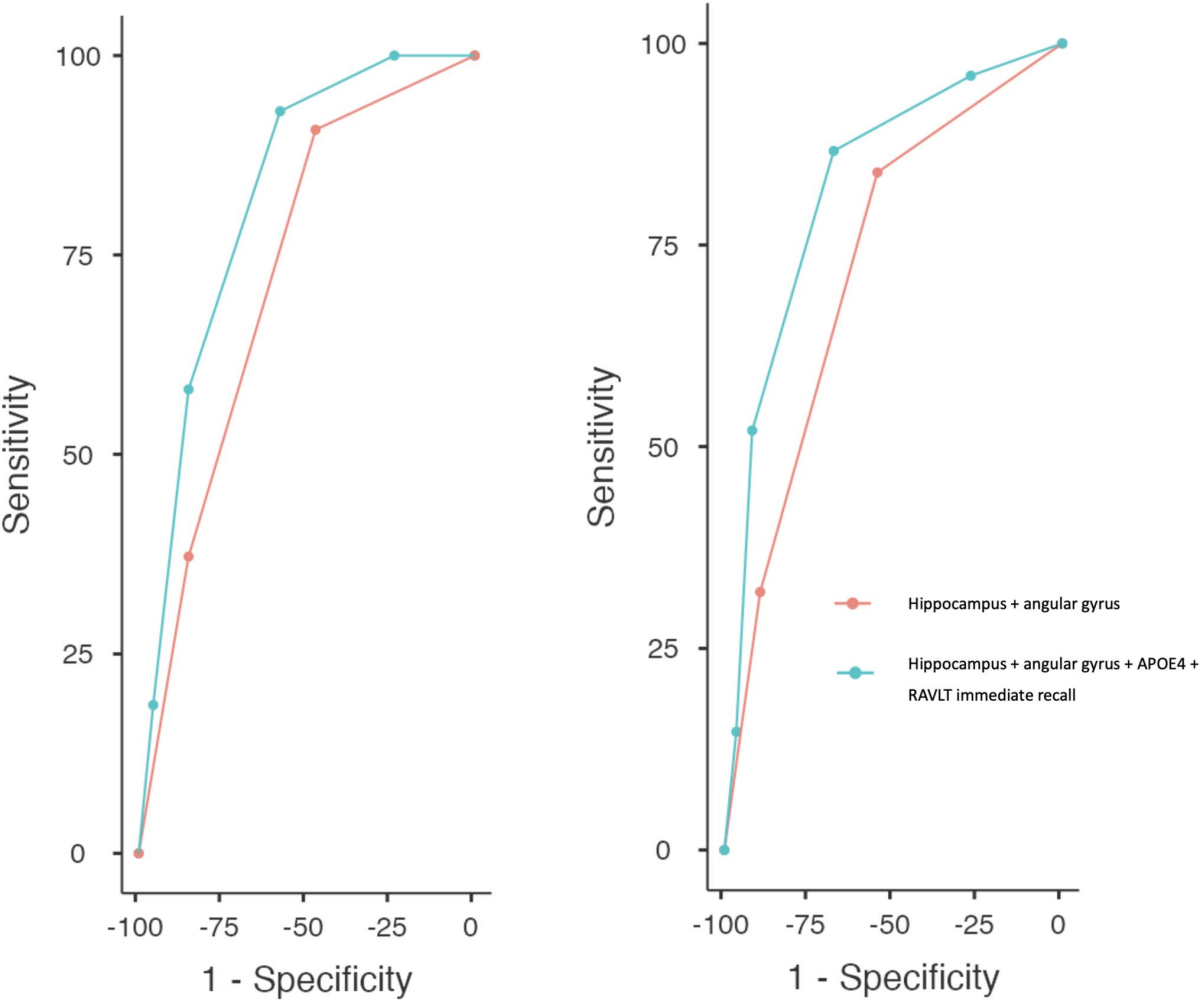
ROC curves of the model combining hippocampus and angular gyrus and the model comprehensive of neuroimaging, neuropsychological, and genetic data. On the left is the global cohort, and the right in the prediction of the eligibility of anti-Aβ treatment

The predictive model, obtained by combining the hippocampus and angular gyrus volumes, RAVLT immediate recall, and APOE4 status as described in the “Statistical analysis” section, achieved the highest accuracy. Notably, the AUC was 0.82, and a threshold value of ≥ 2 predictive score points yielded an optimal sensitivity of 86.6% but a lower specificity of 67.6%. In contrast, a threshold value of ≥ 3 predictive score points resulted in a remarkable specificity of 91.7% but with a lower sensitivity of 52.0% (see Table 4 and Fig. 3).

### Prediction of the eligibility for anti-Aβ treatment

Visual rating scales did not help predict eligibility for anti-Aβ treatment (AUC < 0.55). Automatic rating scales slightly increased predictivity, with the best performance achieved by the computed MTA (AUC = 0.65). In addition, volumetric analysis represented the most accurate tool. The hippocampal volume reached an AUC of 0.70, and the angular gyrus reached a value of 0.67.

When the two regions were combined into the predictive model, the AUC remained at 0.70 with a sensitivity of 90.9% and a specificity of 43.7% at the cutoff ≥ 1. In contrast, the cutoff > 2 displayed a low sensitivity of 38.6% and a higher specificity of 81.9%.

Further incorporation of APOE4 status and the results of the RAVLT immediate test into the global predictive model yielded the best accuracy with an AUC of 0.81 (Table 4, Fig. 3).

## Discussion

Our study demonstrates that automatic neuroimaging assessment, utilizing computed rating scales and volumetric analysis, slightly enhances the prediction of amyloid-CSF status compared to visual rating scales in a naturalistic memory clinic. The integration of volumetric analysis with the results obtained from neuropsychological tests and APOE genotype status achieved good overall performance in the global cohort and in predicting eligibility for anti-Aβ treatment.

One primary objective of our work was to evaluate and compare the predictive performance of atrophy rating scales, commonly used in clinical settings [32], obtained through visual assessment by neuroradiologists and automatic methods. The automatic evaluation demonstrated significantly higher performance in this comparison, particularly for parietal-GCA (AUC = 0.69) and MTA (AUC = 0.66) scores. This improvement may be attributed to the subjectivity of visual assessment and the intrinsic capability of automatic methods to continuously assess brain atrophy, obtaining even decimal results [22], leading to a particularly relevant role in a cohort of patients with relatively low mean age, characterized by potential subtle brain change.

The amyloid-positive and amyloid-negative groups show no differences in white matter changes measured by the Fazekas score. As a result, neither visual nor automated Fazekas score serves as a reliable predictive tool in our cohort. This finding may be related to the high prevalence of early-onset patients and the possibly lower percentage of individuals showing mixed degeneration types (AD-related and vascular degeneration).

While not attaining optimal accuracy and primarily restricted to a research context, the volumetric analysis of MRI confirms superior performance than visual rating scales, showing its potential application in the clinic.

The volume of the hippocampus (AUC = 0.68) and angular gyrus (AUC = 0.69) resulted as the main areas in predicting amyloid-CSF status, consistent with their roles in AD neuropathology [33, 34], and previously highlighted pattern of atrophy in early-onset AD [35, 36], respectively.

These regions demonstrated contrasting sensitivity and specificity, with the normalized hippocampal volume (cutoff < 6.94 ml) displaying higher sensitivity (79.3%) and the angular gyrus (cutoff < 7.80 ml) having higher specificity (84.3%). This finding underscores that, in a cohort characterized mainly by early-onset cognitive impairment (mean age = 59.8 years) and potentially high prevalence of minimal atrophy and hippocampal-sparing subtypes [37], the hippocampus may be affected by volume loss. However, its low specificity may be related to the potential impact of psychiatric and non-AD-related disorders (such as frontotemporal dementia) on affecting hippocampal areas [38, 39]. Contrary to expectations, given the epidemiological characteristics of the cohort, the angular gyrus was not affected by atrophy in a high proportion of patients within the AD continuum. At the same time, it remained a specific hallmark of AD-related pathology.

The contrasting sensitivity and specificity in predicting the amyloid status allowed us to combine them in predictive model scores. As expected, combining hippocampus and angular gyrus volumes enhanced the accuracy (AUC = 0.72).

Even though automated imaging analysis, which uses rating scales and brain volumetry, has proven to be more effective than visual and subjective assessment of atrophy, the predictive results are currently not yet highly accurate in determining CSF-amyloid status.

To provide memory clinics with better tools for screening patients who may require more advanced diagnostic methods, such as CSF analysis, we have combined volumetric neuroimaging data with neuropsychological tests and the APOE4 genotype.

Integrating the hippocampus and angular gyrus volumes with the RAVLT immediate recall test and APOE4 status into a comprehensive model achieved good/excellent accuracy (AUC = 0.82). This result indicates that even in a naturalistic and heterogeneous cohort comprising primarily early-onset cognitive impairment, the integration of clinical, genetic, and neuroradiological data can achieve high accuracy in the diagnostic process. Furthermore, this finding highlights the significance of neuroimaging in improving predictive values, surpassing the results obtained with RAVLT and APOE4 data alone (AUC 0.82 compared to 0.74).

The model proposes different cutoff points applicable in diagnosing patients with cognitive disorders and helps to avoid unnecessary and expensive biological assessments (such as CSF or amyloid PET) in some cases.

A sensitivity of 96% if at least one of the four items (hippocampal and angular gyrus volume, RAVLT performance, and APOE genotype) is altered indicates that patients with a score of 0 are at low risk of being amyloid-positive. This finding is critically important, as it represents a primary objective of this study. Developing tools that maximize sensitivity is essential to ensure that patients who might benefit from further biological assessment are not overlooked. In this context, patients with normal values across all the four parameters evaluated by the predictive model can be considered at substantially low risk of underlying AD neuropathology.

On the other hand, in cases of pathological results in all four items, the specificity for CSF-amyloid status positivity reaches high values (96.4%).

Furthermore, we analyzed how structural neuroimaging can predict the eligibility of anti-Aβ treatment.

The future clinical implementation of disease-modifying treatment needs tools that may help clinicians accurately perform more advanced investigations, such as amyloid PET and CSF analysis [40].

Our results showed that visual and automatic scales and volumetric analyses are slightly less effective in predicting eligibility for anti-Aβ treatment than in predicting amyloid status.

Volumetric assessment obtained the best accuracy, even if it reached only a discrete performance, particularly considering the hippocampus volume (AUC = 0.70) and the predictive model adding the volume of the angular gyrus (AUC = 0.70). In our opinion, the superior predictivity of eligibility of volumetric assessment over computed rating scales lies in its intrinsic capability to intercept the subtle brain changes characterizing the early stages of the disease [41]. Indeed, most of the disease-modifying trials in the field of AD employed selective criteria, comprising the exclusion of the most severe patients beyond the mild AD stage [31] who, being in the latter stage of the disease, may be affected by high neurodegeneration and a typical pattern of atrophy.

Although imaging assessment showed overall low accuracy for determining eligibility for anti-Aβ treatment (AUC ≤ 0.7), when combined with the RAVLT immediate recall score and the APOE4 gene status in the predictive model, we achieved good/excellent accuracy (AUC = 0.81). Given the increasing demand for diagnostic assessment in the future, this finding could be of pivotal importance [42]. Therefore, we provide tertiary clinics, which are more likely available for automatic neuroimaging analysis and tests to investigate APOE4 status, with simple model points to select patients eligible for advanced diagnostic assessments such as amyloid PET and CSF investigations [40].

Our model is not meant to replace the biological diagnosis of AD but rather to assist in the diagnostic process. The increasing demand for diagnosis due to the disease’s growing prevalence is expected to prolong the time needed to obtain a biological diagnosis. This is especially true considering the need for qualified personnel and facilities to perform lumbar punctures [43]. By utilizing available assessments in tertiary memory clinics, such as neuroimaging, neuropsychological testing, and APOE4 genotyping, we can optimize patient selection to identify low- and high-risk groups for AD-related changes effectively.

This finding is important as prior studies indicated that standard assessments from routine clinical practice have high diagnostic accuracy for AD [44]. However, these studies typically concentrated on patients in advanced stages and late-onset cognitive disorders. As a result, they may not adequately address the variability and diagnostic challenges present in younger patient groups, where our model demonstrates strong performance.

Adding data on plasma biomarkers to the model might represent a pivotal point to enhance global accuracy, considering their promising role in AD diagnosis [45, 46]. Blood biomarkers have the potential to revolutionize early AD diagnostics. However, this approach faces limited widespread availability due to high equipment costs, varying methodologies, and a need for standardized cutoff values. In addition, while results have been promising in specific cohorts, further analysis is needed in more representative outpatient settings also to explore the potential correlation of these biomarkers with comorbidities [47].

We acknowledge the suboptimal predictive efficacy of neuroimaging data in our cohort, even with volumetric data. Two primary explanations arise: first, our study, conducted in a real-world memory clinic, explored atrophy differences not between patients in the AD continuum and healthy controls but among patients with various cognitive disorders, where neuroimaging structural differences may be less prominent.

Furthermore, automated volumetric methods differ from voxel-based morphometry analyses. Although the latter is highly accurate, its time-consuming nature and challenges in integration into routine clinical practice should be considered.

Our study has several limitations. The primary limitation is the relatively small proportion of patients within the AD continuum compared to the group with a negative amyloid status. Although this contrasts with the previously reported frequency of AD in memory clinics [48], the relatively young age of our cohort might explain this finding, considering the increasing prevalence of AD with age [49].

Furthermore, the high proportion of early-onset patients in our cohort may make it not fully representative of other memory clinics, given that in Sweden, primary care often diagnoses and manages dementia cases with standard presentations. Meanwhile, the Karolinska Clinic is a reference for early diagnosis of individuals younger than 70 years in the entire Stockholm region. The small number of patients over 65 (= 44) limits the ability to conduct analyses with sufficient statistical robustness in the late-onset group. However, the benefits of automatic imaging compared to visual assessment may still be significant. In older populations, the typical atrophy pattern of the disease often shows a higher frequency of mesial temporal atrophy instead of parietal atrophy, which could affect the predictive accuracy of both visual ratings and volumetric analyses in this cohort [50].

Due to the study’s characteristics, the atrophy scales were evaluated by expert neuroradiologists during routine clinical practice; therefore, inter-rater agreement values are not available. However, a recent study showed that such agreement among experienced personnel is high for each visual rating scale [16].

Lastly, complete MRI reports to evaluate potential microbleeds, which contraindicate anti-Ab treatments, were unavailable.

However, our study is the first to investigate the predictive role of neuroimaging features, assessed manually and automatically, both independently and in conjunction with genetic and neuropsychological data, in determining amyloid status and eligibility for anti-amyloid treatment in an extensive case study representative of a real-world memory clinic setting.

## Conclusion

Our study indicates that the automated brain MRI assessment may offer added benefits compared to subjective assessment in quantifying brain atrophy at a tertiary memory clinic. By combining volumetric data with neuropsychological and genetic information, we can predict amyloid-CSF status and eligibility for anti-Aβ treatment with a high level of accuracy within the naturalistic and heterogeneous context of a memory clinic.

## Data Availability

Professor Kivipelto’s research team is open to requests for data collected in this study. Study plan (including the research question, planned analysis, and data required) will be evaluated on a case-by-case basis. Shared data will encompass the data dictionary and de-identified data only. Analysis will be conducted in collaboration with Professor Kivipelto’s team. Access is subject to the GEDOC legal framework. An access agreement will be prepared.
